# Survival Rate and Cervical Bone Loss of Dental Implants Placed in Regenerated Areas with Free Iliac Graft

**DOI:** 10.30476/DENTJODS.2022.90184.1474

**Published:** 2023-03

**Authors:** Gholamreza Shirani, Mahboubeh Hashemi Nasab, Shahryar Bashiri, Sheida Kordi

**Affiliations:** 1 Dept. of Oral and Maxillofacial Surgery, Shariati Hospital, Tehran University of Medical Sciences, Tehran, Iran; 2 Oral and Maxillofacial Surgeon, Tehran, Iran; 3 Postgraduate Student, Dept. of Oral and Maxillofacial Surgery, Shariati Hospital, Tehran University of Medical Sciences, Tehran, Iran

**Keywords:** Alveolar bone loss, Dental implant, Survival rate, Augment bone graft

## Abstract

**Statement of the Problem:**

For many years, practitioners have been encountered with dental rehabilitation of atrophic jaws. Among many of alternatives, free iliac graft can be a reasonable and also problematic choice to be accomplished.

**Purpose:**

The aim of this study was to evaluate the implant survival rate and bone loss in implants inserted in reconstructed jaws with free iliac graft.

**Materials and Method:**

In this clinical trial study, twelve patients that underwent bone reconstruction with free iliac graft were included in this retrospective study. The patients underwent surgery over a 6-year period from September 2011 to July 2017. Panoramic images were taken immediately after implant insertion and at the follow-up session. The parameters that were assessed included implant survival rate, bone level changes, and surrounding tissue conditions.

**Results:**

One hundred and nine implants were placed in eight female and four male patients, of which 65 (59.6%) were inserted in the reconstructed maxilla and 44 (40.3%) in the reconstructed mandible. The interval between the reconstruction surgery and follow-up session was 28.75 months and the mean interval between implant insertion and the follow-up session was 21.75 months, ranging from 6 to 72 months. The total average of crestal bone resorption was 2.44 mm (range: 0 to 5.43 mm).

**Conclusion:**

This study found that rehabilitation of atrophic jaws with dental implants placed in free iliac graft was associated with acceptable marginal bone loss, survival rate, satisfaction, and esthetic results among the patients.

## Introduction

Many techniques have been advocated for alveolar ridge augmentation [ 1 ]. Different causes such as trauma, tooth extraction, periodontal disease and several pathological conditions lead to jaw atrophy. However, restoration of the oral function, aesthetic aspects, and mastication of atrophic jaws remain a challenge in dental implantology [ 1 ]. Resorption of jaws is lifelong, irreversible, chronic, and cumulative. The greatest amount of resorption occurs during the first year with the most rapid rate in the first three months [ 1 ]. Autogenous bone has been considered as the gold standard for grafting procedures and an ideal bone substitute [ 2 ]. All three crucial characteristics, including osteoconductive, osteoinductive, and osteogenic properties are combined in autogenous bone graft with no risk of disease transmission [ 2 ].

Osteogenesis is of particular importance. It has been shown that autogenous bone grafts such as iliac crest grafts can induce osteogenesis due to a high proportion of bone marrow and human growth factors as well as a large number of living undifferentiated cells [ 3 ]. However, limitations of autografts including limited donor sites, postoperative morbidity, unpredictable resorption, complexity of the surgical procedure, and increased operation time have been debated [ 2 ]. 

Extraoral donor sites such as the iliac crest, tibia, or calvarium have shown acceptable potency for reconstruction of atrophic ridges, although free iliac crest graft remains the first choice due to its sufficient quantity and harvest safety [ 4 - 5 ]. Among extra oral donor sites, it has been postulated that iliac crest and tibial grafts have higher resorption rates as they originate from endochondral ossification [ 6 ]. 

Placement of osseointegrated dental implants following maxillomandibular iliac crest grafting significantly promotes all aspects of oral function in partially or completely edentulous patients [ 7 ]. Furthermore, the results have shown the low morbidity and high reliability of free iliac grafts in preprosthetic alveolar ridge rehabilitation. A few follow-up studies have evaluated the long-term outcomes of patients undergoing free iliac graft reconstruction; however, it is critical to assess the long-term clinical outcomes for a thoughtful treatment planning.

The purpose of this study was to evaluate the survival rate, success, and marginal bone loss of dental implants placed in nonvascularized iliac bone graft after reconstruction of atrophic jaws.

## Materials and Method

**Figure 1:**
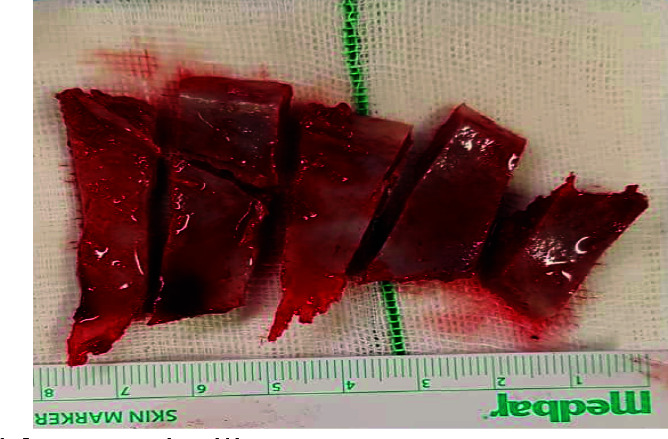
Bone blocks harvested from anterior iliac crest

**Figure 2:**
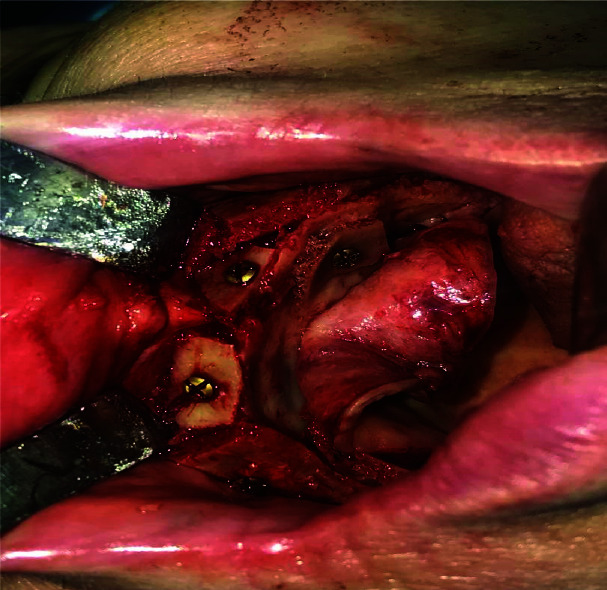
Intraoperative photograph showing bone blocks fixation

**Figure 3:**
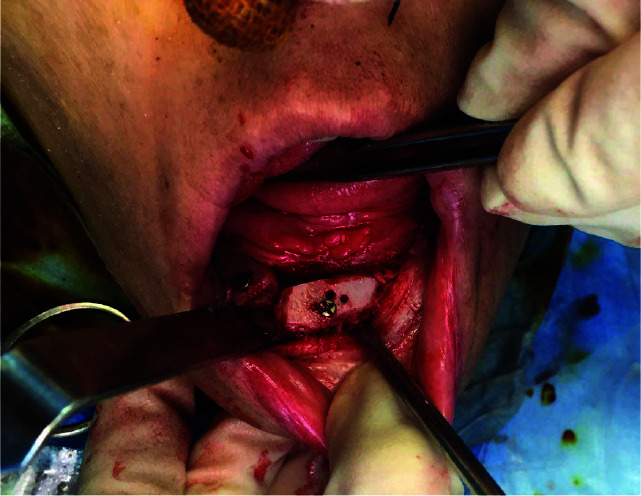
Intraoperative view of iliac graft fixation via titanium screws

**Figure 4:**
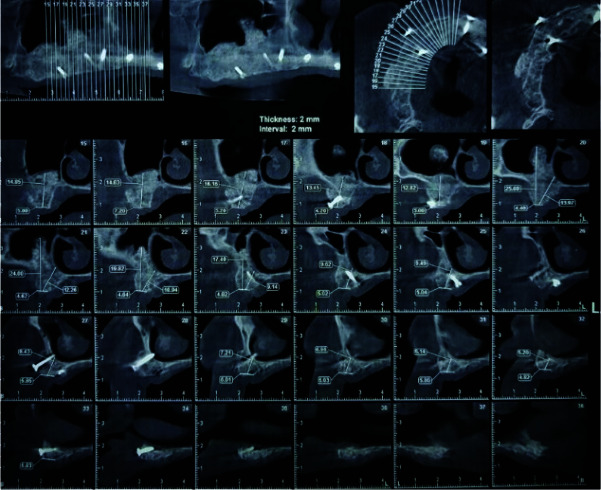
Cone beam computed tomography (CBCT) 6 months postoperatively

**Figure 5:**
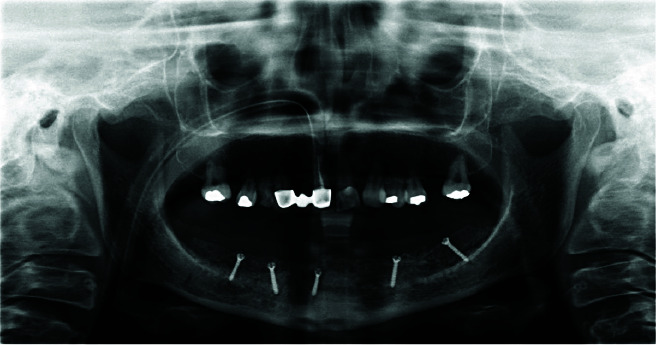
Panoramic view immediately after reconstruction surgery

Twelve patients who underwent jaw reconstruction with free iliac graft over a 6-year period from September 2011 to July 2017 were included in this retrospective study. The inclusion criteria were meeting the diagnostic criteria for jaw reconstruction with autogenous iliac graft prior to implant insertion, no contraindication to surgery, no history of systemic diseases or drug abuse, and a good socioeconomic status to attend follow-up sessions. Exclusion criteria were any history of diabetes, use of immunosuppressive medications (like steroids), alcoholism, osteoporosis, and history of previous surgery due to pathological lesions or unsuccessful reconstruction. Informed consent was obtained from all patients and the Ethics Committee of Tehran University of Medical Science (IR.TUMS.DENTISTRY.REC.1397. 100) approved the study design. Corticocancellous blocks were harvested from the anterior iliac crest and fixed to mandible and maxilla by means of titanium mini screws (Figures 1-3). The gaps between the autogenous blocks were filled by allograft bone substitute. A single surgeon performed all surgeries. None of the patients had nerve transposition surgery. All patients were evaluated six months after reconstruction using panoramic and cone beam computed tomography (CBCT) images (Figures 4-5).

 Following reconstruction, 109 endosseous implants were inserted at the level of crestal bone in both jaws. Two different brands were used in the present study, including Dentium (Dentium Co, Seoul, Korea) in 11 patients and Dentis (Dentis Co, korea) in one patient (three implants). 

**Figure 6:**
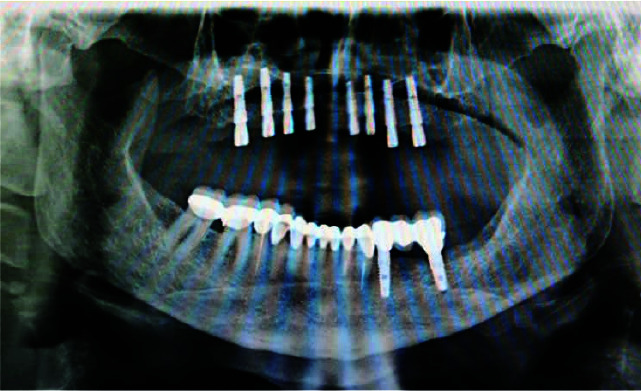
Panoramic view after implant insertion

**Figure 7:**
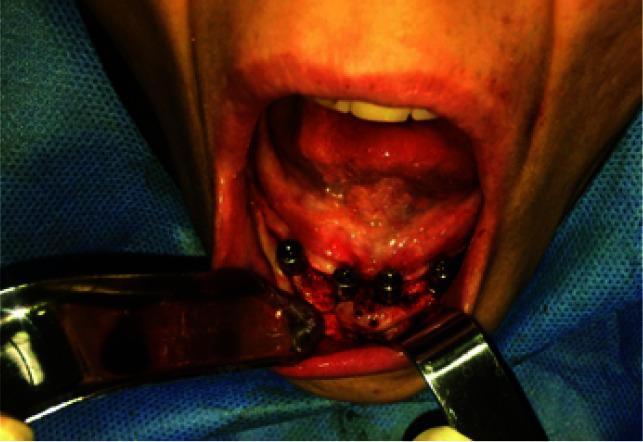
Intraoperative view of implant insertion surgery

**Figure 8:**
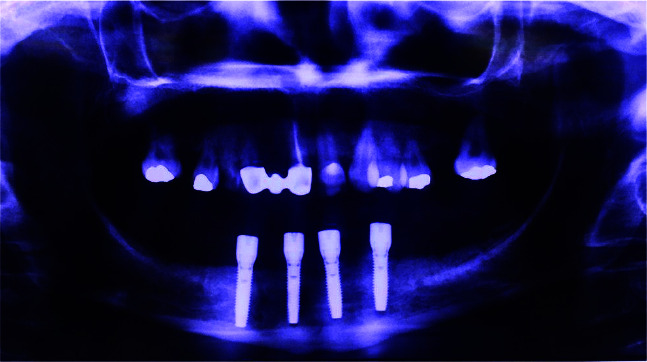
Panoramic radiograph immediately after implant surgery

On average, each patient received 9.08 dental implants. Panoramic images were obtained immediately after implant placement and at the time of prosthetic rehabilitation (Figures 6-7). The reason behind choosing panoramic images was the retrospective nature of the study; all patients already had preoperative panoramic views. All patients were recalled for follow-up annually. Panoramic radiographs were taken at the time of annual follow-ups and the same dentist (Figure 8) assessed all parameters. The measured parameters included implant bone level change, survival rate, implant mobility, peri-implant tissue condition, stability, and function of implant- supported prosthesis.

During the follow-up examination, inflammation, presence of any swelling, quality of peri implant soft tissue, color, consistency, contour, and mobility were checked. Peri-implant mucosal health was defined as pink, firm, and well-adapted gingival tissue. Peri-implant probing was performed to assess the condition and the level of soft and hard tissues.

**Figure 9:**
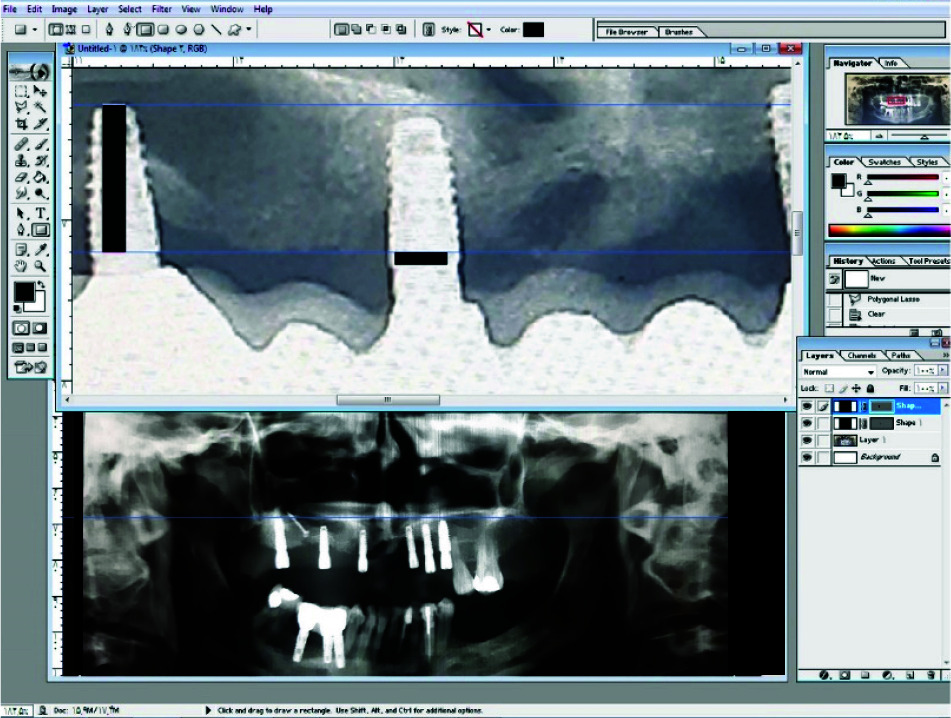
Bone resorption measurement via Adobe Photoshop cc 2019

Patients were asked about aesthetics, phonetics, mastication, and function of implant-supported prosthesis. Cervical bone loss was recorded by comparing panoramic radiographs at the time of implant placement and final follow-up session (Figure 9). The bone level of mesial and distal areas were measured by recording the distance between the most coronal part of the implant neck and the most coronal level of the direct bone-to-implant contact. Dimensional distortion was corrected by knowing the actual length of the implants that were recorded in patients’ files.

 X=B×C÷A, in which A= length of implant in radiograph, B= actual length of implant, C= distance between the most coronal part of implant neck and the most coronal level of direct bone-to-implant contact in radiograph, and X= actual distance between the most coronal part of implant neck and the most coronal level of direct bone-to-implant contact.

The peri-implant bone loss level was calculated by reducing the amount of X in images taken immediately after implant placement from the amount of X in follow-up radiographs. Adobe Photoshop CC 2019 software was used to measure the bone level loss in millimeters. 

## Results

**Figure 10:**
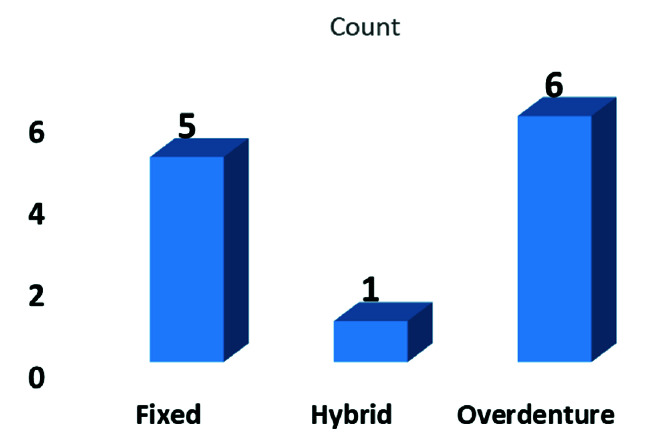
Prosthesis type distribution

Twelve patients including 8 females and 4 males with a mean age of 53.33 years were included in this study. Three out of 12 patients (25%) were cigarette smokers. Of the 109 dental implants, 65 were inserted in the reconstructed maxilla (59.6%) and 44 in the reconstructed mandible (40.3%). Transient paresthesia was detected in half of the patients (50%). The affected area was the lower lip in three patients and the mid-face area in two patients. Only one patient complained about paresthesia in both upper and lower lip areas. The mean interval between reconstruction time and the follow-up session was 28.75 months and the mean interval between implant placement and the follow-up session 21.75 months ranging from 6 to 72 months. The received prosthetic treatment was categorized as 1. removable overdenture of maxilla and mandible (4 patients), 2. removable overdenture of mandible (2), 3. hybrid fixed prosthesis of maxilla and mandible (1), 4. fixed prosthesis of maxilla and mandible (1), 5. fixed maxillary prosthesis (3), and 6. fixed maxillary prosthesis and mandibular removable overdenture (1) (Figure 10).

Gingival recession was detected in five patients including three males and two females. In total, 14 out of 109 implants (12%) had variable degrees of gingival recession. Bleeding on probing was seen in two patients, including one female and one male, in four out of 109 implants (3.6%). Inflammation was detected in three patients (two females and one male) in three out of 109 implants (2.75%). There were no non-osseointegrated and non-functional implants.

The total average of crestal bone resorption was 2.44 in 12 patients (109 implants) ranging from 0 to 5.43 (Table 1). The spearman rank correlation between age and crestal bone resorption was 58.04%, which was significant except for the age group 57-63, which has been decreased (Table 2). The mean crestal bone resorption in male and female patients was 3.10 and 1.86 mm, respectively. There was a significant correlation between gender and crestal bone resorption (Mann-Whitney test, *p*= 0.0376). The mean crestal bone resorption in three time intervals including 6-18 (74 implants), 18-40 (21 implants) and more than 40 months (14 implants) was 1.88mm, 2.93mm and 3.99mm, respectively. The average crestal bone resorption was 3.11 and 2.29 in smokers and non-smokers respectively, which showed no statistically difference between the two groups (Two-sample Wilcoxon rank-sum (Mann Whitney) test *p*= 0.1655).

**Table 1:** Cervical bone resorption and number of implants

Number of Implants	Cervical Bone Resorption (mm)
21	0-1
41	1-2
23	2-3
10	3-4
6	4-5
8	5 and more

**Table 2:** Age range and average amount of resorption

Average Amount of Resorption (mm)	Age Range (Years)
1.41	36-42
1.92	43-49
3.24	50-56
2.95	57-63
4.37	64-67

Satisfaction with prosthetic rehabilitation was achieved in 10 out of 12 patients, of whom 2 were not completely satisfied.

## Discussion

Different factors including host defense mechanism, recipient bed, graft volume, method of protecting graft after harvesting, sufficient contact of the graft and recipient bed, and integrity of the harvested bone with the atrophic jaws can affect the resorptive process [ 9 ]. This phenomenon is inhibited by physiologic stress, stimulation, and persistent and dynamic loading of prosthetic treatment [ 9 ]. Implant insertion in the reconstructed maxilla and mandible has been advocated to decrease bone resorption. Bone resorption rates of 30-90% have been reported after augmentation in removable denture wearers [ 8 ]. 

The survival rate of dental implants has been reported to be 60-70% by Keller *et al*. [ 10 ]. In the present study, intimate bone graft incorporation with host bone was observed in all 12 patients and the survival rate after a follow-up course (average: 21.75 months with a range of 6-72 months) was 100%, indicating that restoring all aspects of oral function in patients with atrophic jaws can be achieved by reconstruction prior to implant insertion.

In other studies, a cumulative survival rate of 97.2% was also found in patients who underwent implant placement in free iliac graft following mandible segmental resection [ 11 ]. According to a systematic review [ 12 ] and some retrospective studies [ 13 - 15 ], implant insertion in native and augmented bone is associated with equal results, which is consistent with our findings.

Nkenke *et al*. [ 16 ] found that although implant survival and success rates following reconstructive procedures were high, the type of implant was more important compared to the type of bone graft. However, similar to the present study, they were not able to find a significant relationship between the type of implant and cumulative success and survival rates.

The bone loss level was measured in many studies, which all found bone loss levels ranging from 0.5 to 3 mm in the first year of function. After the first year of implant insertion, the resorption rate was not significant and could be considered relatively negligible in most cases [ 16 - 19 ].

Quiles *et al*. [ 17 ] found an average bone loss of 1.74mm and 1.08mm in the maxilla and mandible in the first year, respectively. However, Kondell *et al*. [ 18 ] reported higher rates of bone loss (2.15mm) in patients re-constructed with rib graft in the first year. The amount of cervical bone loss following implant insertion in rec-onstructed jaws varies significantly in different studies. Nystorm *et al*. [ 19 ] found that the mean bone loss in implants inserted following iliac crest graft was 2.23mm and 2.60mm in male and female patients, respectively. The survival rate of Straumann bone level implants inserted in calvarial and ramus graft was 100% in a study by Chiapasco *et al*. [ 20 ] Moreover, in this study; the bone loss level was 0.41 and 0.52mm in carlvarial and ramus graft respectively, which was less than other studies. The results of the present study revealed that the healthy peri-implant tissue was present in all cases and no implant failure was observed because of severe gingival hyperplasia or bone resorption. There was no mobility, no sign of pathologic lesions, and no hard tissue complications.

Of the different success criteria, annual vertical bone loss less than 0.2 mm is the most important one, which was observed in 77 implants in our study (70.64%). In addition, vertical bone loss less than 3 mm was found in 85 implants in the follow-up session, indicating the high success rate of this procedure. It can be construed that reconstruction of severely atrophic jaws with iliac crest free graft before implant insertion is a safe and predictable alternative.

## Conclusion

Within the limits imposed by the variety of initial clinical situations, limited number of patients and implant samples, and the type of reconstruction performed, this study found that autogenous ilium bone grafting was an effective means to reconstruct atrophic jaws following bone atrophy. This technique demonstrated an excellent prognosis for restoration of the oral function in patients as evidenced by the high survival rate of the implants and low marginal bone loss placed in the reconstructed areas. Implants placed in free iliac grafts had an excellent prognosis among implant-supported prostheses. Moreover, there was no significant bone loss around implants in free iliac grafted bone except for one implant. The patients expressed an acceptable level of satisfaction with the restoration of their oral function; furthermore, implants placed in the graft were stable and no complications occurred in the peri-implant tissue. The patients regained masticatory function with acceptable aesthetic results.

## Conflict of Interest

The authors declare that they have no conflict of interest.
